# EXPLORING NOVEL DETERMINANTS OF EXERCISE BEHAVIOR: A LAGGED EXPOSURE-WIDE APPROACH

**DOI:** 10.1093/geroni/igae098.0529

**Published:** 2024-12-31

**Authors:** Harold Lee, Eric Kim, David Conroy, Tyler VanderWeele

**Affiliations:** Penn State, University Park, Pennsylvania, United States; University of British Columbia, Vancouver, British Columbia, Canada; The Pennsylvania State University, University Park, Pennsylvania, United States; Harvard T. H. Chan School of Public Health, Boston, Massachusetts, United States

## Abstract

Many middle-aged to older adults do not engage in regular exercise at all, despite its importance for healthy aging. Extensive research grounded in behavioral and social science theories has identified numerous determinants of exercise. However, few studies used an exposure-wide approach, a data-driven exploratory method that is particularly useful for identifying determinants. We used data from 13,771 participants in the Health and Retirement Study (HRS), a diverse, national panel study of adults aged >50 in the United States, to evaluate 62 candidate predictors of exercise participation. Candidate predictors were drawn from the following domains: health behaviors, physical health, psychological well-being, psychological distress, social factors, and work. We used Poisson regression with robust error variance to individually regress exercise in the outcome wave (t2:2014/2016) on baseline candidate predictors (at t1;2010/2012) controlling for all covariates in the previous wave (t0;2006/2008). Some health behaviors (e.g., ever exercising), physical health conditions (e.g., physical functioning limitations and lung disease), psychological factors (e.g., health mastery, purpose in life, and positive affect), and social factors (e.g., helping others, religious service attendance, and volunteering) were robustly associated with increased subsequent exercise. Among factors related to psychological distress, perceived constraints stood out as a factor in reducing exercise. To conclude, we identified potentially novel exercise determinants, such as purpose in life and helping others, that have not been captured using a theory-driven approach. Future studies validating these findings in older adults are needed.

